# Identification of a Novel lncRNA LNC_001186 and Its Effects on CPB2 Toxin-Induced Apoptosis of IPEC-J2 Cells

**DOI:** 10.3390/genes14051047

**Published:** 2023-05-06

**Authors:** Kaihui Xie, Qiaoli Yang, Zunqiang Yan, Xiaoyu Huang, Pengfei Wang, Xiaoli Gao, Shuangbao Gun

**Affiliations:** 1 College of Animal Science and Technology, Gansu Agricultural University, Lanzhou 730070, China; xiekh@st.gsau.edu.cn (K.X.); yangql0112@163.com (Q.Y.); yanzunqiang@163.com (Z.Y.); huanghxy100@163.com (X.H.); wangpf815@163.com (P.W.); gxl18892@163.com (X.G.); 2 Gansu Research Center for Swine Production Engineering and Technology, Lanzhou 730070, China

**Keywords:** CPB2 toxin, piglet diarrhea, lncRNAs, IPEC-J2 cells, apoptosis

## Abstract

The *Clostridium perfringens* (*C. perfringen*) beta2 (CPB2) toxin produced by *C. perfringens* type C (*CpC*) can cause necrotizing enteritis in piglets. Immune system activation in response to inflammation and pathogen infection is aided by long non-coding RNAs (lncRNAs). In our previous work, we revealed the differential expression of the novel lncRNA LNC_001186 in *CpC*-infected ileum versus healthy piglets. This implied that LNC_001186 may be a regulatory factor essential for *CpC* infection in piglets. Herein, we analyzed the coding ability, chromosomal location and subcellular localization of LNC_001186 and explored its regulatory role in CPB2 toxin-induced apoptosis of porcine small intestinal epithelial (IPEC-J2) cells. RT-qPCR results indicated that LNC_001186 expression was highly enriched in the intestines of healthy piglets and significantly increased in *CpC*-infected piglets’ ileum tissue and CPB2 toxin-treated IPEC-J2 cells. The total sequence length of LNC_001186 was 1323 bp through RACE assay. CPC and CPAT, two online databases, both confirmed that LNC_001186 had a low coding ability. It was present on pig chromosome 3. Cytoplasmic and nuclear RNA isolation and RNA-FISH assays showed that LNC_001186 was present in the nucleus and cytoplasm of IPEC-J2 cells. Furthermore, six target genes of LNC_001186 were predicted using *cis* and *trans* approaches. Meanwhile, we constructed ceRNA regulatory networks with LNC_001186 as the center. Finally, LNC_001186 overexpression inhibited IPEC-J2 cells’ apoptosis caused by CPB2 toxin and promoted cell viability. In summary, we determined the role of LNC_001186 in IPEC-J2 cells’ apoptosis caused by CPB2 toxin, which assisted us in exploring the molecular mechanism of LNC_001186 in *CpC*-induced diarrhea in piglets.

## 1. Introduction

Piglet diarrhea caused by pathogenic microbes leads to massive piglet mortality, thereby seriously endangering the healthy development of pig farming [1]. The bacterium *Clostridium perfringens* (*C. perfringens*) type C (*CpC*) is the primary cause of piglet diarrhea. Studies have shown that *CpC* rapidly colonizes and multiplies in the intestines of pigs, thereby producing toxins that damage the intestinal mucosa epithelium and increase intestinal epithelial permeability, causing necrotizing enterocolitis (NE) [2,3]. *CpC* infects the host primarily by producing α and β (CPB1 and CPB2) toxins [4,5]. Among them, *C. perfringens* beta2 (CPB2) toxin was separated from a *CpC*-infected piglet that passed away from NE and it is a novel *CpC*-produced toxin [6]. This toxin can cause NE and enterotoxemia in humans and animals, especially piglets and CPB2 toxin exerts a toxic damaging effect on CaCo-2 and I407 cells [6,7,8]. Gao et al. [9] and Luo et al. [10] reported that cell barrier function was impaired and apoptosis and inflammatory responses were triggered by CPB2 toxin in porcine small intestinal epithelial (IPEC-J2) cells. Here, we constructed an in vitro cell injury model using IPEC-J2 cells exposed to CPB2 toxin in order to investigate the possible mechanism of action of *CpC* infection in piglets with diarrhea and to discover effective molecular targets for therapy.

Long noncoding RNAs (lncRNAs) are a class of non-coding RNAs with more than 200 nt and tissue specificity, but without reading frame (ORF) or protein-coding ability [11]. Extensive studies have indicated that lncRNAs have multiple roles, such as chromatin remodeling, mRNA splicing and editing and transcriptional regulation, and are involved in several biological processes including apoptosis, cell growth and carcinogenesis [12,13,14,15]. Meanwhile, lncRNAs act as a key regulator in the infection process of pathogenic microorganisms affecting animals. For instance, Wu et al. [16] discovered that knocking down the lncRNA FUT3-AS1 increased IPEC-J2 cells’ resistance to *Escherichia coli* (*E. coli*) F18, suggesting that it might serve as a molecular biomarker of piglets having *E. coli* F18 infection resistance. Gao et al. [17] demonstrated that lncRNA IALNCR suppressed bovine viral diarrhea virus (BVDV)-induced apoptosis of bovine kidney (MDBK) cells, promoted BVDV replication and was crucial for regulating host antiviral innate immunity against BVDV infection. Tanuj et al. [18] found that the lncRNA TCONS_00076023 targeted cja-miR-612 to regulate the inflammatory response to Peste des petits ruminant virus (PPRV)-induced B lymphocytes in marmosets. Zhang et al. [19] demonstrated that downregulated LNC_000397 significantly attenuated antiviral interferon-stimulated genes expression, such as MX dynamin-like GTPase 1 (*MX1*), interferon-stimulated gene 15 (*ISG15*) and radical S-adenosyl methionine domain containing 2 (*RSAD2*), which in turn restrained the replication of Porcine Reproductive and Respiratory Syndrome virus (PRRSV). In our previous study, a novel lncRNA LNC_001186 was identified to be significantly upregulated in *CpC*-infected ileum tissue in piglets with diarrhea [20], suggesting its possible involvement in regulating *CpC*-infected process in piglets. Therefore, in this study, the sequence characteristics and subcellular location of LNC_001886 were first analyzed, and its expression in IPEC-J2 cells exposed to CPB2 toxin was examined. Subsequently, functional overexpression and deletion were used to examine LNC_001886’s impact on IPEC-J2 cells apoptosis induced by CPB2 toxin, providing a conceptual framework for further investigation into the molecular mechanism by which LNC_001886 contributes to *CpC*-induced diarrhea in piglets.

## 2. Materials and Methods

### 2.1. Animal Sample Collection

We established a *CpC*-induced piglet diarrhea model as described in our previous method [21]. Piglets were fed with 1 × 10^9^ CFU/mL of *CpC* bacterial solution for the susceptible group (IS, *n* = 5), and control piglets (IC, *n* = 5) were fed with sterile culture solution. Ileum, duodenum, jejunum, lung, heart, spleen, kidney, lymph, liver and stomach tissues were collected from the piglets and quickly stored at −80 °C in liquid nitrogen. All animal experiments were carried out in accordance with the regulations established by the Experimental Animal Center’s Ethics Committee of Gansu Agriculture University (Approval No. 2006-398).

### 2.2. IPEC-J2 Cells Culture and Damage Model Establishment

IPEC-J2 cells (BNCC, Beijing, China) were cultured in a humidified 5% CO_2_ environment at 37 °C in a DMEM/F12 (Hyclone, Logan, UT, USA) medium supplemented with 1% penicillin/streptomycin (Hyclone) and 10% fetal bovine serum (FBS; Gibco, Waltham, MA, USA). The cell damage model was built by treating IPEC-J2 cells with 20 μg/mL of CPB2 toxin for 24 h. The steps of CPB2 toxin preparation and the specific infection process have been described previously [9,10].

### 2.3. RNA Isolation and cDNA Synthesis

Total RNA was extracted from the tissues and IPEC-J2 cells by the TRIzol reagent (Leagene, Beijing, China). The concentration of RNA was measured using an ultra-micro spectrophotometer (Thermo Fisher Scientific, Waltham, MA, USA), and the integrity of the RNA was examined with 1% agarose gel. Reverse transcription kits (Accurate Biotech, Changsha, China) were used to convert RNA into cDNA, which was then stored at −20 °C.

### 2.4. Rapid Amplification of cDNA Ends (RACE)

Reverse transcription of RNA from the ileum tissue of healthy pigs yielded 5′ and 3′ RACE cDNA templates for PCR amplification, as per the manufacturer’s instructions for the GeneRacer^®^ Kit (Invitrogen, Carlsbad, CA, USA). The recovered and purified amplification products were ligated into the pGM-T vector, used to convert DH5α competent cells (TIANGEN, Beijing, China) and then coated onto Luria–Bertani (LB) solid medium. Single colones were picked for sequencing analysis. The primers were synthesized by TsingkeBiotechnology Co., Ltd. (Xi’an, Shanxi, China), and the sequences are presented in Table 1.

### 2.5. Cytoplasmic/Nuclear RNA Isolation Experiment

The PARIS™ Kit (Invitrogen) was employed to extract IPEC-J2 cytoplasmic and nuclear RNA. IPEC-J2 cells (1 × 10^7^) exhibiting excellent growth were collected, and RNA was extracted based on the manufacturer’s specifications. The cDNA that resulted from the transcription of RNA was frozen at −20 °C. In IPEC-J2 cells, RT-qPCR was used to detect cytoplasmic and nuclear LNC_001186 expression.

### 2.6. RNA-FISH

The IPEC-J2 cells (1 × 10^4^ cells/well) were seeded into 24-well plates and fixed with 4% paraformaldehyde for 15 min after they were stuck to the wall. The cells were subsequently stained using the FISH kit’s instructions (GenePharma, Shanghai, China). LNC_001186 distribution was visualized under a fluorescent inverted microscope (Olympus IX71, Tokyo, Japan) after the staining was completed. An LNC_001186 probe was synthesized by (GenePharma Co., Ltd., Shanghai, China) and the sequence was 5′-TACTTAGGCTTTGAAAGGGCAGTGGGC-3′.

### 2.7. Bioinformatics Analysis

The *cis* and *trans* method was employed to predict the target genes of LNC_001186. Protein-coding genes were selected as *cis* target genes within 10–100 kb upstream and downstream of LNC_001186. The Pearson correlation coefficient (|r| > 0.95) was used to determine the target genes of LNC_001186 in the *trans*. The ceRNA regulatory network centered on LNC_001186 was constructed using our previous lncRNA RNA-seq (PRJNA399620) and miRNA RNA-seq (GSE130471) data of the ileum tissue of *CpC*-infected piglets. miRNAs with binding sites to LNC_001186 and negatively correlated expression were screened. The target genes of miRNAs were predicted using miRanda (http://www.microrna.org/microrna/home.do, accessed on 20 January 2021), PicTar (https://pictar.mdc-berlin.de/, accessed on 20 January 2021) and TargetScan (https://www.targetscan.org/vert_72/, accessed on 20 January 2021) softwares.

### 2.8. Overexpression Vector Construction and siRNA Synthesis

According to the specifications of the GeneRacer® Kit (Invitrogen, Carlsbad, CA, USA), RNA from the ileum tissue of healthy piglets was reverse transcribed into 5′ and 3′ RACE cDNA templates for PCR amplification. In order to construct the LNC_001186 overexpression vector, the PCR products were recovered, purified, and then cloned into the pcDNA3.1(+) vector (Promega, Madison, WI, USA). Sequencing and double digestion allowed the effective overexpression vector to be identified and termed as pc-LNC_001186. LNC_001186 siRNA was designed and synthesized by GenePharma Co., Ltd. Table S1 provides a list of the sequences for the LNC_001186 siRNA and the negative control (NC).

### 2.9. Cell Transfection

After the IPEC-J2 cells reached 80–90% confluence, pc-LNC_001186 (800 ng), LNC_001186 siRNA (150 nM) and their respective NCs [pcDNA3.1(+) vector (800 ng) and si-NC (150 nM)] and Opti-MEM (Gibco) culture medium were co-transfected into the cells using the Lipofectamine™ 2000 reagent (Invitrogen). After 24 h of successful transfection, the cells were collected, RNA was extracted and transfection efficiency was determined using RT-qPCR. Compared with the NC groups, an overexpression efficiency of more than 10-fold and knockdown efficiency of more than 60% were considered as successful transfection.

### 2.10. CCK-8 Assay

In 96-well plates, IPEC-J2 cells (1 × 10^3^ cells/well) were seeded, and once they were in good condition the transfection and CPB2 toxin treatment were carried out. After 24 h, each well was treated with 10 μL of CCK-8 solution (Absin, Shanghai, China) that was gently mixed. The plates were incubated at 37 °C for 3 h. The absorbance was then measured at 450 nm using a multifunctional enzyme marker (Molecular Devices in Silicon Valley, CA, USA). Blank and control wells were established at the same time.

### 2.11. Flow Cytometry

Cell apoptosis was determined using the Annexin V-FITC Cell Apoptosis Detection Kit (Beyotime, Shanghai, China). The IPEC-J2 cells (1 × 10^6^ cells/well) in the logarithmic stage were seeded into 6-well plates. These cells underwent transfection and the CPB2 toxin treatment. The cells were collected after 24 h and centrifuged at 1000× *g* for 5 min. After discarding the supernatant, PBS was used to gently resuspend the cells. The supernatant was discarded after the cells were centrifuged again for 5 mins at 1000× *g*. Subsequently, 200 µL of Annexin V-Fluorescein Isothiocyanate (FITC) and 10 µL of propidium iodide (PI) staining solution were added to the cells and mixed gently. For 15 min, the cells were incubated in the dark and at room temperature. A flow cytometer (Cytek Biosciences, Silicon Valley, USA) was used to assess the apoptosis of the cells.

### 2.12. LDH Activity Test

The supernatant from each group of IPEC-J2 cells was collected and then centrifuged at 2000 rpm for 5 min, and the supernatant was retained. Then, 20 µL of the supernatant was added to a 96-well enzyme labeling plate, which included assay and control wells. Blank and standard wells were also established. The samples were spiked according to the specifications of the LDH activity assay kit (Beyotime), and absorbance was tested at 450 nm on a multifunctional enzyme marker (Molecular Devices).

### 2.13. RT-qPCR

LncRNA expression was detected using RT-qPCR on a LightCycler 480 II (Roche Applied Science, Mannheim, Germany) through the SYBR^®^ Green Premix Pro Taq HS qPCR Kit (Accurate). An internal reference was the *GAPDH* gene. The 2^−ΔΔCt^ method was used to determine the relative expression of genes [22]. The RT-qPCR primers were synthesized by TsingkeBiotechnology Co., Ltd., and the sequences are listed in Table 2.

### 2.14. Western Blot

RIPA lysate (Solarbio, Beijing, China) containing protease inhibitors was used to extract IPEC-J2 cells protein. The BCA Protein Concentration Assay Kit (Beyotime) was used to calculate the protein concentration. Following a 10 min incubation at 100 °C, the protein underwent denature. Electrophoresis using 10% SDS-PAGE was then carried out. Membranes made from polyvinylidene difluoride (PVDF) were used for transferring the proteins. The membranes were blocked with 5% skimmed milk for 2 h at room temperature after being rinsed once with tris-buffered saline with Tween (TBST). The primary antibodies (Bioss, Beijing, China) were then added and diluted, and incubated at 4 °C overnight. The membranes were washed with TBST for five minutes the next day, and the procedure was repeated three times. The membranes were subjected to diluted secondary antibody (IgG/HRP) incubation at room temperature for 1.5 h, followed by three TBST washes. Eventually, the enhanced chemiluminescence (ECL) kit (Proandy, Xian, China) was used to observe protein bands. Table S2 provides specific information of antibodies.

### 2.15. Statistical Analysis

The one-way ANOVA was performed on the test data of the two groups by using SPSS 21.0 (IBM Corp, Armonk, NY, USA). Moreover, the data were denoted as mean ± SD, and each assay was repeated 3 times independently. *p* < 0.05 showed prominent difference; *p* < 0.01 specified highly prominent difference. The GraphPad Prism 6.0 software (GraphPad Inc., La Jolla, CA, USA) was used to visualize the obtained data.

## 3. Results

### 3.1. Pig LNC_001186 Characteristics

**Figure 1:**
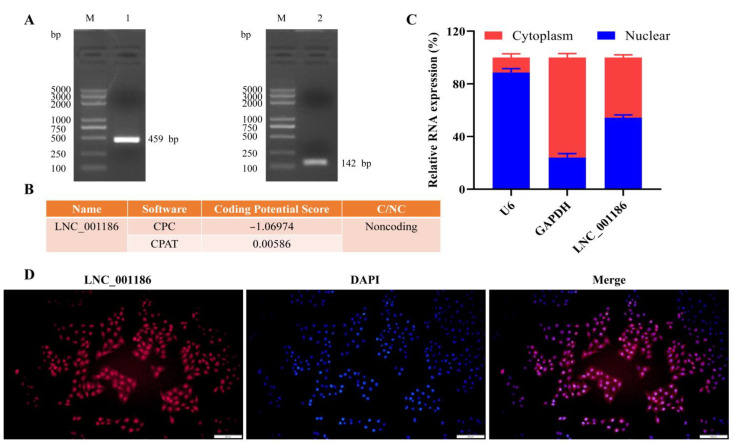
Full-length sequence and subcellular localization of LNC_001186. (**A**) The 5′ RACE and 3′ RACE PCR amplification products. M, DL5000 DNA Marker; 1, 5′ RACE PCR amplification product; 2, 3′ RACE PCR amplification product. (**B**) Online databases CPC and CPAT predicted the coding ability of LNC_001186. (**C**) Expression of LNC_001186 in IPEC-J2 cells determined through RT-qPCR. (**D**) Distribution of LNC_001186 in IPEC-J2 cells determined through RNA-FISH, scale bar = 200 μm.

The full-length LNC_001186 sequence was obtained by RACE PCR amplification. Sequences of 459 bp and 142 bp were obtained through 5′ and 3′ RACE, respectively, and the total length of the LNC_001186 sequence was 1323 bp (Figure 1A). The sequences are shown in Figure S1. LNC_001186 is located on pig chromosome 3. CPC (http://cpc2.gao-lab.org/, accessed on 11 December 2020) and CPAT (https://wlcb.oit.uci.edu/cpat/, accessed on 11 December 2020) predicted that LNC_001186 had a low-coding ability (Figure 1B). Subsequently, the cytoplasmic and nuclear RNA separation experiment and RNA-FISH were performed to detect LNC_001186 expression sites in IPEC-J2 cells. LNC_001186 was simultaneously expressed in the IPEC-J2 cell nucleus and cytoplasm (Figure 1C,D).

### 3.2. LNC_001186 Regulatory Network Construction

**Figure 2:**
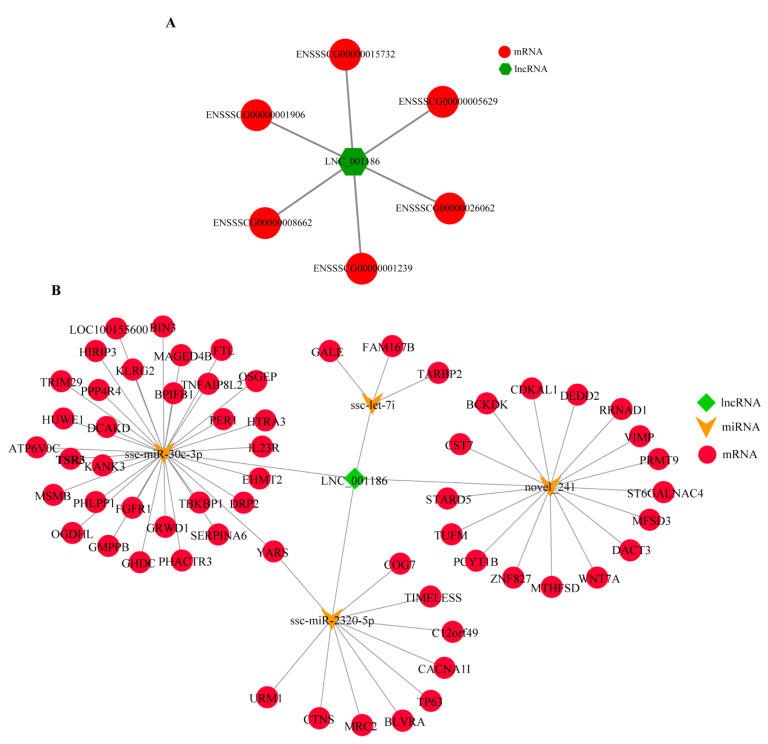
LNC_001186 regulatory network. (**A**) LNC_001186-mRNA interacting network. (**B**) LNC_001186-miRNA-mRNA ceRNA regulatory network.

The function of lncRNAs is highly dependent on its subcellular location. LNC_001186 was expressed simultaneously in the IPEC-J2 cell nucleus and cytoplasm, which suggests that LNC_001186 regulates the expression of target genes both pre- and post-transcriptionally. By combining the previous lncRNA RAN-seq data (PRJNA399620), we predicted that the six target genes of LNC_001186 were differentially expressed in IS and IC groups’ ileum tissue by using *cis* and *trans* approaches (Figure 2A). In the meantime, based on the previous lncRNA RAN-seq and miRNA RAN-seq data (GSE130471), the ceRNA regulatory network with LNC_001186 was constructed. As shown in Figure 2B, LNC_001186 may be the adsorption sponge of differentially expressed ssc-let-7i, ssc-miR-30c-3p, ssc-miR-2320-5p and novel_241 in *CpC*-infected piglets’ ileum tissue and in healthy piglets.

### 3.3. LNC_001186 Expression Pattern Analysis

**Figure 3:**
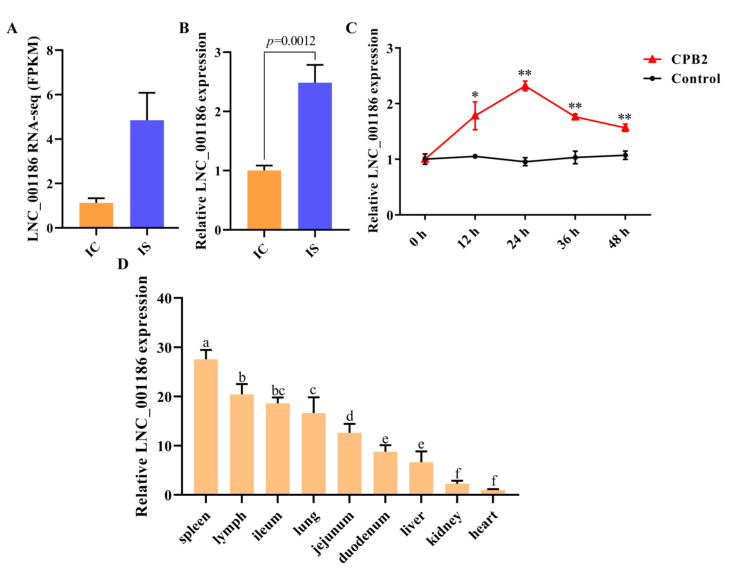
LNC_001186 was upregulated in IPEC-J2 cells treated with CPB2 toxin. (**A**) LNC_001186 RNA-seq expression. (**B**) LNC_001186 expression in healthy piglets’ ileum tissue (IC) and piglets with diarrhea (IS) determined through RT-qPCR (*n* = 3). (**C**) LNC_001186 expression in CPB2 toxin-treated IPEC-J2 cells determined through RT-qPCR (*n* = 3). (**D**) LNC_001186 tissue expression profile (*n* = 3). * *p* < 0.05, ** *p* < 0.01. Different lowercase letters indicate *p* < 0.05.

Our previous RNA-seq results [20] revealed that LNC_001186 expression was higher in the ileum tissue of the IS group compared to the IC group (Figure 3A). The RNA-seq findings were confirmed by the RT-qPCR results (Figure 3B). After that, using RT-qPCR in IPEC-J2 cells, the LNC_001186 expression was evaluated at 12, 24, 36 and 48 h after CPB2 toxin treatment. As depicted in Figure 3C, CPB2 toxin-treated IPEC-J2 cells had observably higher levels of LNC_001186 expression, with a peak at 24 h. Additionally, RT-qPCR was employed to ascertain LNC_001186 expression in different tissues of the piglets. According to the findings, LNC_001186 expression was greatest in the spleen and higher in the lymph, followed by the ileum, lung, jejunum and duodenum, and it was lowest in the liver and kidney, whereas it was barely expressed in the heart (Figure 3D).

### 3.4. LNC_001186 Improved CPB2 Toxin-Treated IPEC-J2 Cell Viability

**Figure 4:**
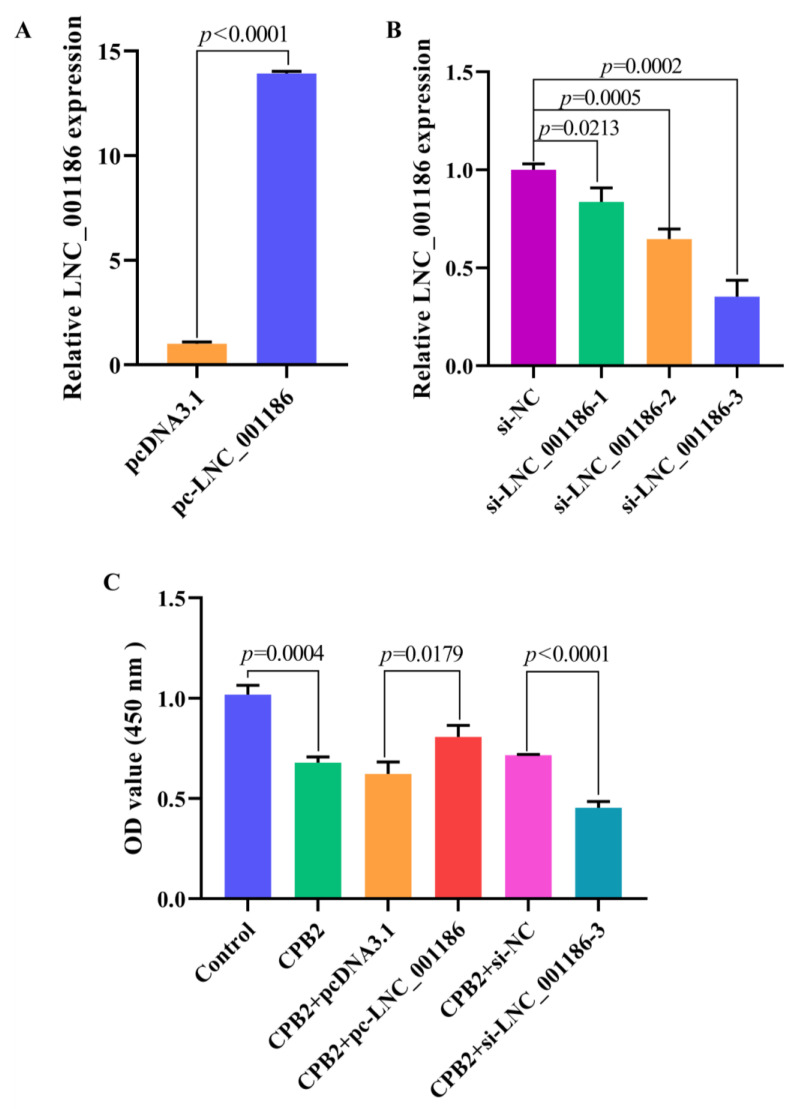
Elevated LNC_001186 enhanced IPEC-J2 cell viability treated with CPB2 toxin. (**A**) pcDNA3.1- and pc-LNC_001186-transfected IPEC-J2 cells. RT-qPCR was used to test the LNC_001186 overexpression efficiency (*n* = 3). (**B**) Transfection of si-NC and si-LNC_001186 into IPEC-J2 cells and detection of the LNC_001186 knockdown efficiency through RT-qPCR (*n* = 3). (**C**) CCK-8 assay for cell viability (*n* = 3).

We transfected the cells with pc-LNC_001186, LNC_001186 siRNA and their corresponding NCs to examine the LNC_001186 effect on CPB2 toxin-treated IPEC-J2 cells’ viability. In comparison to the pcDNA3.1 group, LNC_001186 expression was 14 times higher in the pc-LNC_001186 group (Figure 4A). We designed three different LNC_001186 siRNAs. As displayed in Figure 4B, LNC_001186 expression was the lowest after transfection with si-LNC_001186-3, and the knockdown efficiency was 65%. The CCK-8 assay was used to determine the effect of LNC_001186 on IPEC-J2 cell viability. The results manifested that the CPB2 toxin suppressed cell viability. LNC_001186 overexpression enhanced the IPEC-J2 cells’ viability when treated with CPB2 toxin, while LNC_001186 knockdown repressed the viability of the injured cells. The aforementioned results revealed that LNC_001186 improved IPEC-J2 cell viability treated with CPB2 toxin.

### 3.5. LNC_001186 Suppressed CPB2 Toxin-Induced IPEC-J2 Cell Apoptosis

**Figure 5:**
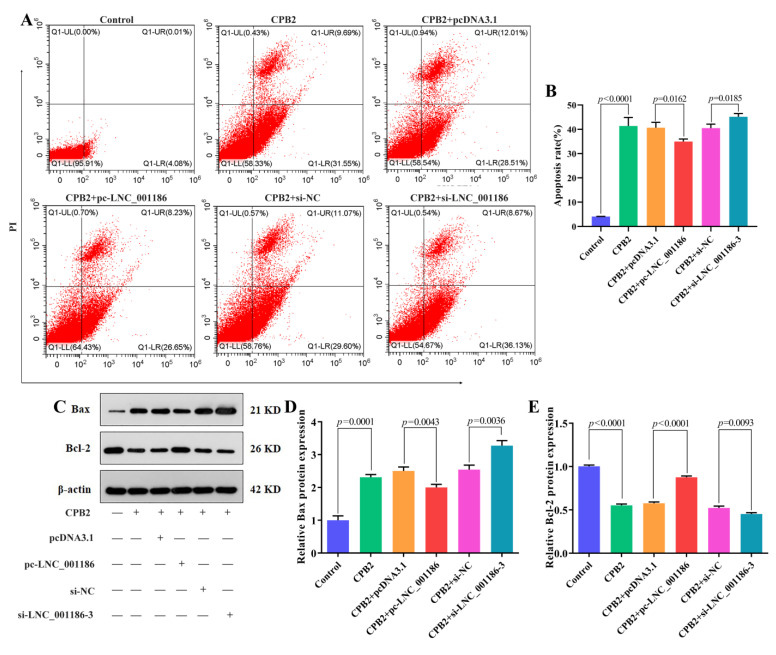
LNC_001186 overexpression suppressed CPB2 toxin-caused IPEC-J2 cell apoptosis. (**A**) Apoptosis detection through flow cytometry. (**B**) Proportion of apoptotic cells. (**C**) Bax and Bcl-2 protein bands. (**D**) Bax relative protein expression (*n* = 3). (**E**) Bcl-2 relative protein expression (*n* = 3).

To determine the impact of LNC_001186 on IPEC-J2 cells’ apoptosis caused by CPB2 toxin, flow cytometry and Western blot were employed. Results from the flow cytometry showed that the CPB2 toxin promoted IPEC-J2 cells’ apoptosis and that LNC_001186 overexpression prevented this apoptosis. On the other hand, LNC_001186 knockdown facilitated apoptosis (Figure 5A,B). Meanwhile, Western blot results indicated that LNC_001186 upregulation depressed the expression of CPB2 toxin-induced the expression of pro-apoptotic protein Bax and accelerated the expression of anti-apoptotic protein Bcl-2, whereas LNC_001186 knockdown aggrandized Bax expression and suppressed Bcl-2 expression (Figure 5C–E). The aforementioned results suggested that elevated LNC_001186 expression repressed apoptosis of CPB2 toxin-treated IPEC-J2 cells.

### 3.6. LNC_001186 Alleviated CPB2 Toxin-Induced IPEC-J2 Cell Cytotoxicity and Inflammation

**Figure 6:**
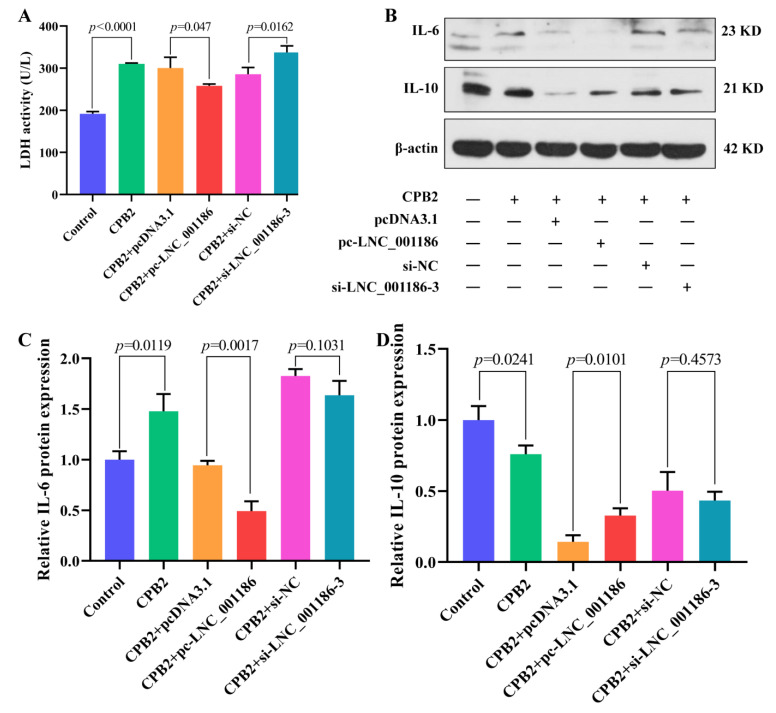
LNC_001186 overexpression attenuated CPB2 toxin-induced cytotoxicity and inflammation in IPEC-J2 cells. (**A**) LDH activity (*n* = 3). (**B**) IL-6 and IL-10 protein bands. (**C**) IL-6 relative protein expression (*n* = 3). (**D**) IL-10 relative protein expression (*n* = 3).

The LDH kit was employed to evaluate IPEC-J2 cell cytotoxicity. As displayed in Figure 6A, the CPB2 toxin enhanced LDH activity in the IPEC-J2 cells, and pc-LNC_001186 transfection repressed LDH activity in damaged cells, whereas si-LNC_001186-3 transfection improved LDH activity. The effect of LNC_001186 on CPB2-induced IPEC-J2 cell inflammation was evaluated through Western blot. According to the results, CPB2 toxin induced IL-6 expression and inhibited IL-10 expression. Compared with the pcDNA3.1 group, LNC_001186 overexpression group lowered IL-6 expression and increased IL-10 expression, whereas LNC_001186 knockdown had no remarkable effect on IL-6 and IL-10 expression (Figure 6B–D). These results suggested that high LNC_001186 expression ameliorated CPB2 toxin-induced IPEC-J2 cell cytotoxicity and inflammation. 

## 4. Discussion

Piglet diarrhea is among the major diseases leading to piglet mortality. We here verified that LNC_001186 was upregulated in piglets’ ileum tissue with *CpC*-induced diarrhea, suggesting that LNC_001186 has a role in the disease pathogenesis. Extensive studies have shown that lncRNAs are aberrantly expressed in various pathogenic bacterial infections and play an essential regulatory role. For instance, lncRNA RDUR expression increased in Influenza A virus (IAV)-infected mice and human alveolar epithelial cells and affected IAV replication [23]. The lncRNA NEAT1-2 was upregulated in Hantaan virus (HTNV)-induced mouse bone-marrow-derived macrophages, and when NEAT1-2 was silenced, inflammatory macrophage activation was repressed and HTNV replication was facilitated [24]. Pawar et al. [25] identified that lncRNA MEG3 expression was reduced in *Mycobacteria*-infected macrophages, and lncRNA MEG3 knockdown induced autophagy. LncRNA ZEB1-AS1 expression was upregulated in *Chlamydia trachomatis* infection, and the silencing of ZEB1-AS1 increased apoptosis of persistently infected cells [26]. According to this study’s findings, LNC_001186 was upregulated in the ileum tissue of *CpC*-infected piglets with diarrhea and in IPEC-J2 cells induced with CPB2 toxin. LNC_001186 was abundantly expressed in intestinal tissues, which indicated that LNC_001186 is a key regulator during diarrhea in *CpC*-infected piglets. Subsequently, we observed that the full-length LNC_001186 sequence was 1323 bp. It had a low coding ability and was located on pig chromosome 3.

The location of lncRNAs in the cell predominantly determines their function. Nuclear lncRNAs may regulate gene expression pre-transcriptionally, whereas cytoplasmic lncRNAs may have an essential role in post-transcriptional regulation and post-translational modifications [27,28]. For example, the lncRNA Airn, which was localized in the nucleus, was involved in insulin-like growth factor 2 receptor (*IGF2R*) silencing by overlapping with its promoter [29]. The cytoplasmic lncRNA TUG1 regulated mutation in multiple advanced cancers 1 (*PTEN*) expression through binding miRNAs [30]. LNC_001186 was discovered to be present in IPEC-J2 cells’ nucleus and cytoplasm, suggesting that it regulates the target gene both pre- and post-transcriptionally. Based on our previous RNA-seq data, we predicted six target genes of LNC_001186 by using *cis* and *trans* approaches. Meanwhile, we constructed the ceRNA regulatory network centered on LNC_001186 and found that LNC_001186 could bind to ssc-let-7i, ssc-miR-30c-3p, ssc-miR-2320-5p and novel-241 to regulate the expression of their target genes. Among them, let-7i and miR-30c-3p have been proved to bind competitively to multiple lncRNAs. For example, Zhang et al. [31] found that lncRNA TTTY15 knockdown suppressed hypoxia-induced apoptosis and the dysfunction of mitochondrial energy metabolism in cardiomyocytes in vitro through the let-7i-5p/TLR3/NF-kappa B pathway. Zhang et al. [32] demonstrated that the lncRNA MALAT1 attenuated TNF-α-induced endothelial cell pyroptosis through the miR-30c/Cx43 axis.

Related studies have revealed that lncRNAs participate in several diseases and affect cell apoptosis. Upregulated expression of the lncRNA GAS5 restrained cell viability and facilitated apoptosis of breast cancer cells, including triple-negative breast cancer cells [33]. The lncRNA-NEAT1 maintained the growth of hepatocellular carcinoma (HCC) cells and inhibited apoptosis and cell cycle arrest under hypoxic conditions [34]. The downregulated lncRNA AC009948.5 facilitated apoptosis of lung adenocarcinoma cells [35]. We found that in the CPB2 toxin-treated IPEC-J2 cells, LNC_001186 overexpression enhanced cell viability and Bcl2 protein expression, and suppressed Bax protein expression and CPB2 toxin-induced apoptosis. Our results suggest that LNC_001186 is a negative regulator of CPB2 toxin-induced IPEC-J2 cell apoptosis. Furthermore, lncRNAs are involved in some inflammatory damage processes. LDH is present in the cytoplasm of all tissues and cells. In damaged tissues or cells, LDH levels increase, which is a crucial indicator of the inflammatory response [36,37]. Hong et al. [38] revealed that lncRNA NONHSAT217600.1 knockdown attenuated neodymium oxide (Nd_2_O_3_)-induced LDH release and reduced inflammatory damage in 16HBE human bronchial epithelial cells. The lncRNA lnc13 suppressed inflammatory gene expression in macrophages [39]. High lncRNA LASI expression increased *IL-6* and *CXCL8* mRNA expression in airway epithelial cells [40]. LncRNA MEG3 knockdown repressed the expression of inflammatory cytokines in the cerebrospinal fluid and serum of rats with cerebral hemorrhage [41]. In this study, elevated LNC_001186 expression inhibited CPB2 toxin-induced cytotoxicity and IL-6 expression in the IPEC-J2 cells and enhanced IL-10 expression. The results of this study indicate that LNC_001186 inhibited CPB2 toxin-induced apoptosis and inflammatory damage in IPEC-J2 cells and is a positive protective factor during *CpC*-induced piglet diarrhea, and could be used to inhibit *CpC*-induced intestinal damage by overexpressing LNC_001186 in subsequent animal experiments and thereby reduce the occurrence of diarrhea in piglets.

## 5. Conclusions

Overall, lncRNA LNC_001186 was expressed in the cytoplasm as well as the nucleus of IPEC-J2 cells and was upregulated in the *CpC*-infected ileum tissue of piglets and in CPB2 toxin-treated IPEC-J2 cells. LNC_001186 overexpression promoted IPEC-J2 cell viability and inhibited apoptosis and inflammatory damage induced by CPB2 toxin. The study results provide a theoretical basis for further investigation of the molecular regulatory mechanism underlying LNC_001186 in *CpC*-induced piglet diarrhea.

## Figures and Tables

**Table 1:** The 5′ and 3′ RACE PCR amplification primer sequences.

Primers	Sequences (5′ to 3′)
5′ RACE	Reverse1: CTTCATGAACCTGTGGCAAGTGCCTGAAA
5′ RACE	Reverse2: GGAGGGCATGGCATGCATTGCAAAG
3′ RACE	Forward1: CCCATGGCCACGCCAGAGCCTTAA
3′ RACE	Forward2: GGGATGGAACCTGTGTCCTCCTGGATA

**Table 2:** RT-qPCR primer sequences.

Primers	Sequences (5′ to 3′)	Accession No.
LNC_001186	Forward: TCTGCCATCTCATCTATTTCGC	/
	Reverse: GTGGCAAGTGCCTGAAAGAC	
*U6*	Forward: GGAACGATACAGAGAAGATTAGC	NC_000015
	Reverse: TGGAACGCTTCACGAATTTGCG	
*GAPDH*	Forward: AGTATGATTCCACCCACGGC	NM_001206359.1
	Reverse: TACGTAGCACCAGCATCACC	

## Data Availability

The original contributions presented in the study are included in the article/supplementary material; further inquiries can be directed to the corresponding authors.

## Supplementary Materials

The following supporting information can be downloaded at: https://www.mdpi.com/article/10.3390/genes14051047/s1, Table S1: The sequences of LNC_001186 siRNA; Table S2: Antibodies’ specific information. Figure S1: Full-length LNC_001186 sequence.
