# Supplementary material for: Identification of a Novel lncRNA LNC_001186 and Its Effects on CPB2 Toxin-Induced Apoptosis of IPEC-J2 Cells
Source: Genes (Basel). 2023 May 6;14(5):1047. doi: 10.3390/genes14051047 (PMC10218644; doi:10.3390/genes14051047)
Supplement: Supplementary file 1 [file genes-14-01047-s001.zip › Supplementary Tables and Figures.pdf]

Table S1. The sequences of LNC_001186 siRNA

siRNA	sense (5′ to 3′)	antisense (5′ to 3′)
si-LNC_001186-1	GCCCUUUCAAAGCCUAAGUTT	ACUUAGGCUUUGAAAGGGCTT
si-LNC_001186-2	GCCUGCUGUUCUAAACAAATT	UUUGUUUAGAACAGCAGGCTT
si-LNC_001186-3	GUGGUUGUCACUUGAAACUTT	AGUUUCAAGUGACAACCACTT
si-NC	UUCUCCGAACGUGUCACGUTT	ACGUGACACGUUCGGAGAATT

Table S2. Information on antibodies used in this study

Protein	Item No.	Dilution proportion	Company
Bcl-2	bs-4563R	1: 1500	Bioss
Bax	bs-0127R	1: 1500	Bioss
IL-6	bs-4587M	1:1000	Bioss
IL-10	bs-0698R	1:1000	Bioss
β-actin	bs-0061R	1:2000	Bioss
Goat Anti-rabbit IgG/HRP	Bs-0295G-HRP	1:2000	Bioss

Figure S1. Full-length LNC_001186 sequence

Figure S1

Full-length LNC_001186 sequence

CGATGTGTGTGTGTGTGTGTGTGTGTGTGTGTGTGTGGCGGGGGGTGGGGGTGGGGGGG
CTGTGGTTGTCACTTGAAACTGGAGAGGGTTCATTTTAACTATGAAATTTTCAATAAAT
CTGAATTCTCCCTCTAGTTCTCCCCCATGATATTTACATTTACCTTTGCTTTTTCTTTTTTT
TTCAGACACTCTTCGGAAAAAAAACGGTAAATTTTCTGATATCCCATTGCTCTTGATCC
CCTCCGAAGAGACTGGTTCAGCCTCTCATCTTGTCTGCCATCTCATCTATTTCGCTTCTG
TCGGCATGCCTTTCTCTTGCACGTCCTGTCTACGGGCTCCACCTCAATGTTTTGCGTGG
GACTTGTCCCACGTTACTTCTGTCCATTTCTGTTTGCCTACCTCCTCTGCTCTGCCATTCC
TTCTCTTTGCAATGCATGCCATGCCCTCCCAATAAGGACTGGCTTTGCTTCGGTCTTTCA
GGCACTTGCCACAGGTTCATGAAGTTGGTGAGATGCAAAGCATTACGCTGAGAGGGA
GATGGTTTCTCTTCCACGTGTGTTTCTCAAAAACATCCAAACACAGGGGCTCTGTCGG
GACTTCCAAGTCAGAGGGGCCATTCCTGCACAAGGGATAGCTCTTCAAAGACTTAAG
AGATGGGAGATGGCCCCAAAACGGCTCAGTTAATGCCCTCTGCCCACTGCCCTTTCAA
AGCCTAAGTAGTATATGCTATCATCCATCGGTCAAGAGTGTGCCAGGGCGCCCCTCTTC
CACTGGCCTTCCCCCCTGGGAACCAAGTATTCCCAGGTATGAACATGTTACTTCAGTTT
GCCTGCTGTTCTAAACAAAAGCCAGGCCTACCGGTTGTGTCATTTTGTGTGTCTCAGA
GTTCTGAAATGTCAGTTCTCCCTGACGGAGGCTGTTGAAAATTTAGAGCAATCTTCAGT
TCCACATAACACATCTTTTCGGTTCCTTTTGGGTCACTCGCTTTTAGAATGATTAGCTTT
CCTCCCCCGAAGTTAGGTGAGATGGCATTACTAGCCTGTTTTGTTTTGTAAGGGCCACA
CCCACCGGCACAGGGACATTCCCAGGCTAGGGGCTGAATGGGAGCTGTAGCCGCCAG
CCTACACCACAGAAGCAGCAAGGCCGGTACCAGCCACCCCTGCGAACCACACACCA
CAGCCCATGGCCACGCCAGAGCCTTAACCTCCTGAGCGAGGGCCGGGATGGAACCTG
TGTCCTCCTGGATACTCGTCCGATTCACTTCCACTGAGCCATGACGGGAACTCCCAGG
AAAGCCTCGAGTCACATCAAAAAAAAAAAAAAA
