# Supplementary material for: Identification of a Novel lncRNA LNC_001186 and Its Effects on CPB2 Toxin-Induced Apoptosis of IPEC-J2 Cells
Source: Genes (Basel). 2023 May 6;14(5):1047. doi: 10.3390/genes14051047 (PMC10218644; doi:10.3390/genes14051047)

Figure 5C

1, Control; 2, CPB2; 3, CPB2+pcDNA3.1; 4, CPB2+pc-LNC_001186; 5, CPB2+si-NC; 6, CPB2+si-LNC_001186-3

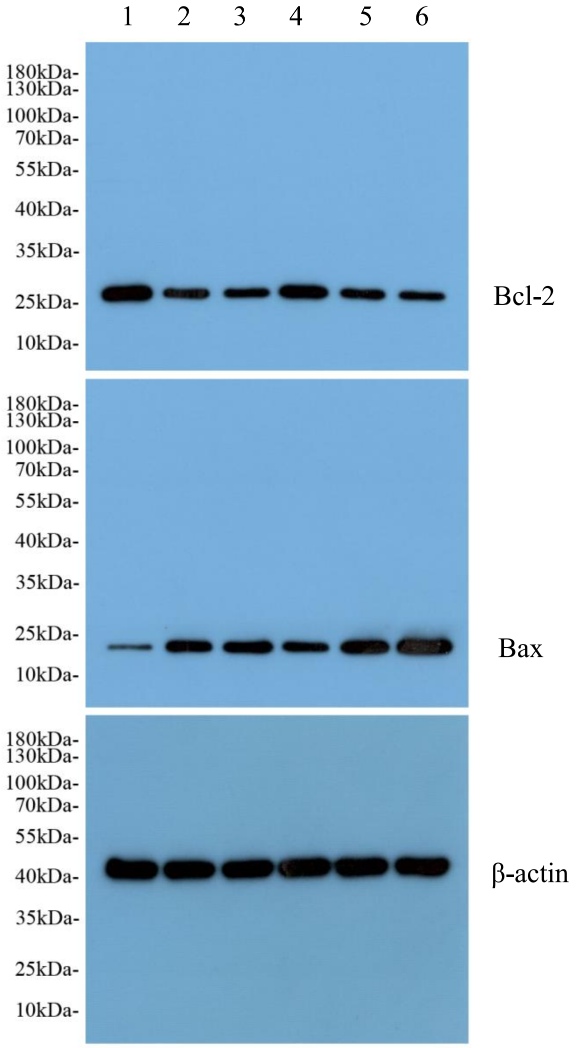

Figure 6B

1, Control; 2, CPB2; 3, CPB2+pcDNA3.1; 4, CPB2+pc-LNC_001186; 5, CPB2+si-NC; 6, CPB2+si-LNC_001186-3

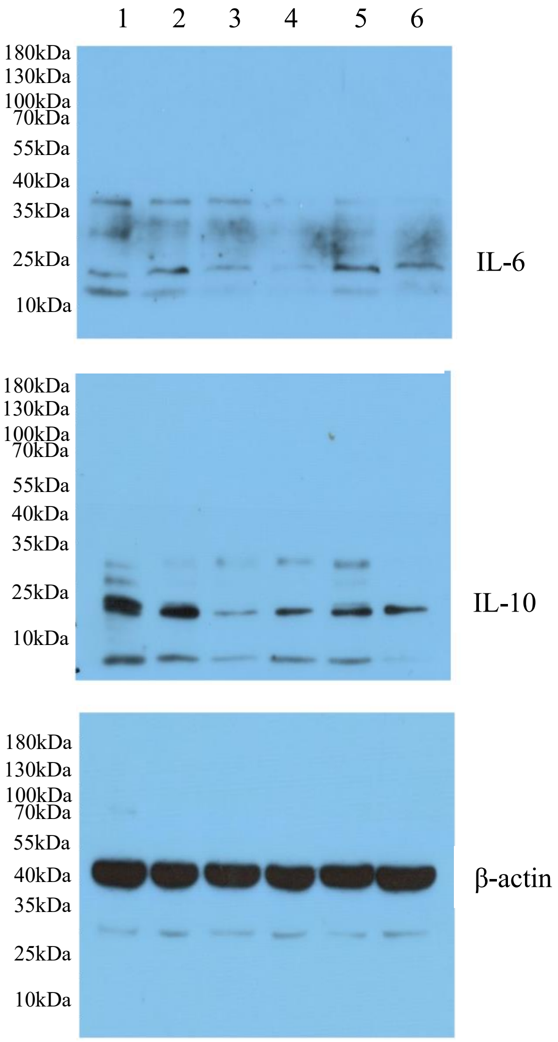

Supplement: Supplementary file 1 [file genes-14-01047-s001.zip › Western blot.pdf]
