# Supplementary figures and images for: Identification of a Novel lncRNA LNC_001186 and Its Effects on CPB2 Toxin-Induced Apoptosis of IPEC-J2 Cells
Source: Genes (Basel). 2023 May 6;14(5):1047. doi: 10.3390/genes14051047 (PMC10218644; doi:10.3390/genes14051047)

**Figure 1D**

LNC\_001186

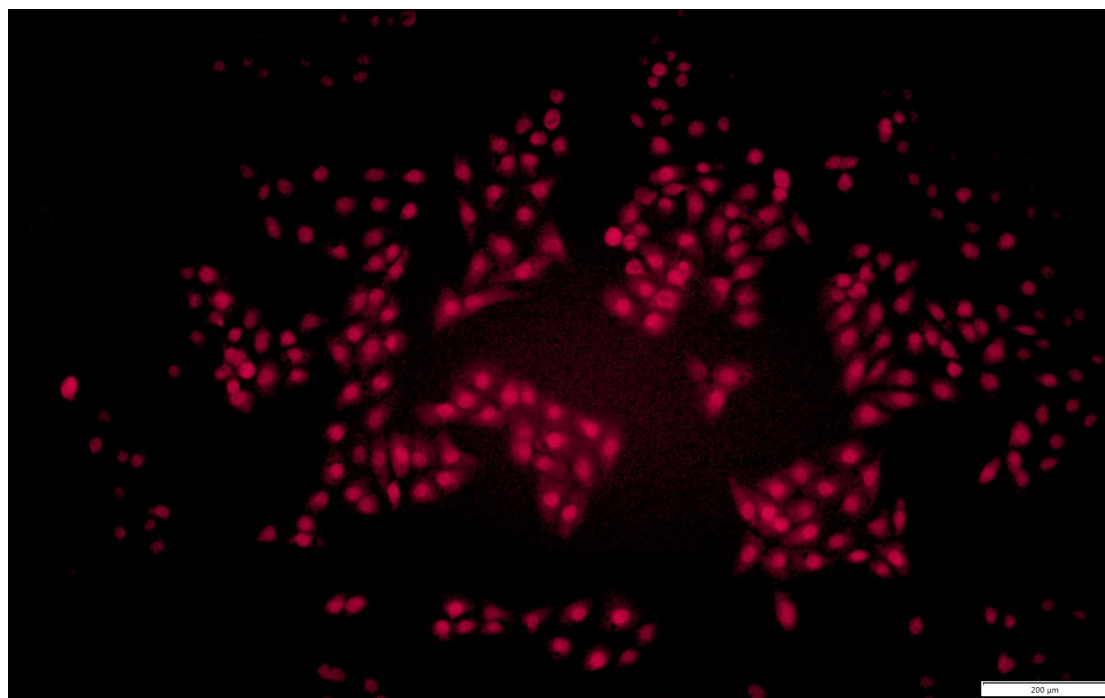

DAPI

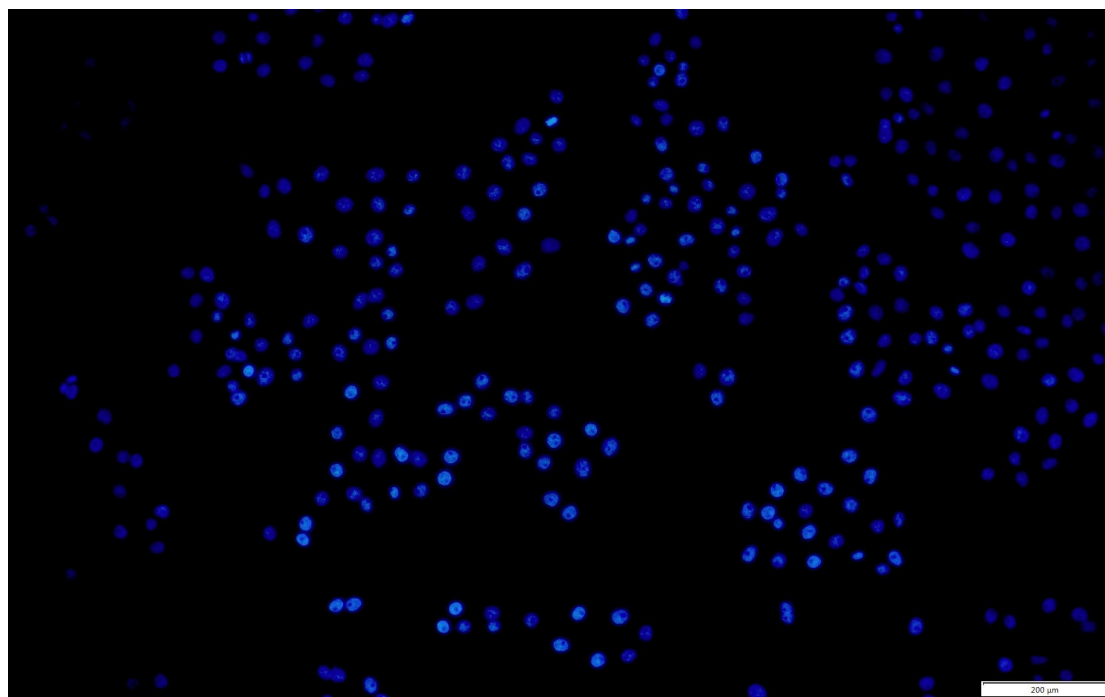

Merge

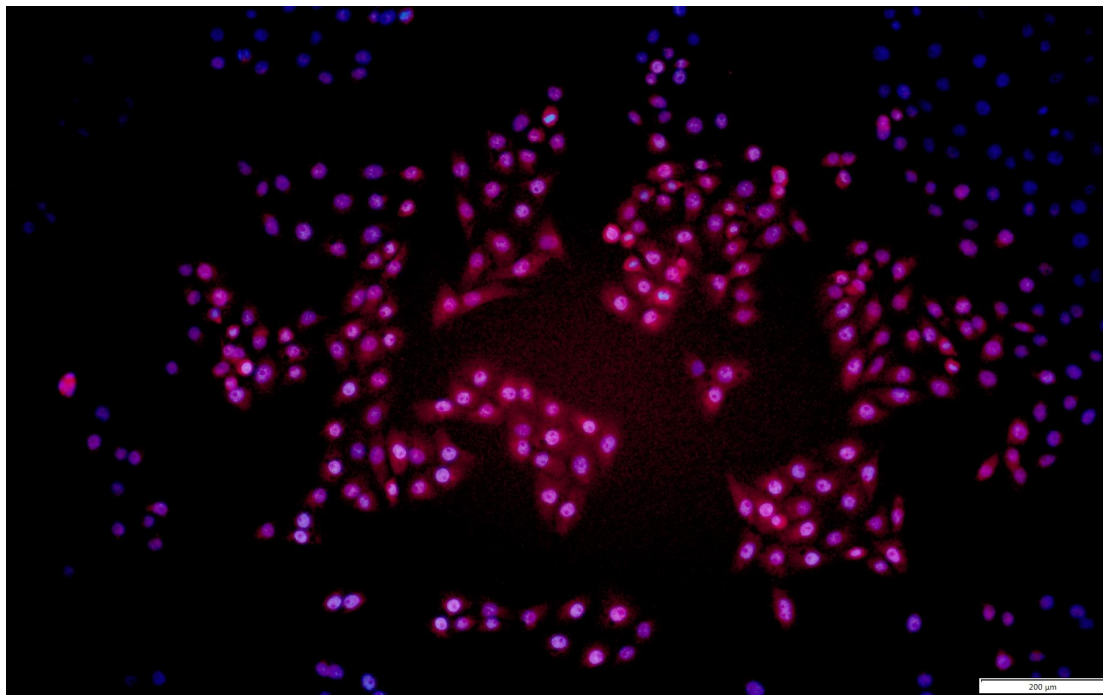

Supplement: Supplementary file 1 [file genes-14-01047-s001.zip › Original image of fluorescence images.pdf]

**Figure 1A**

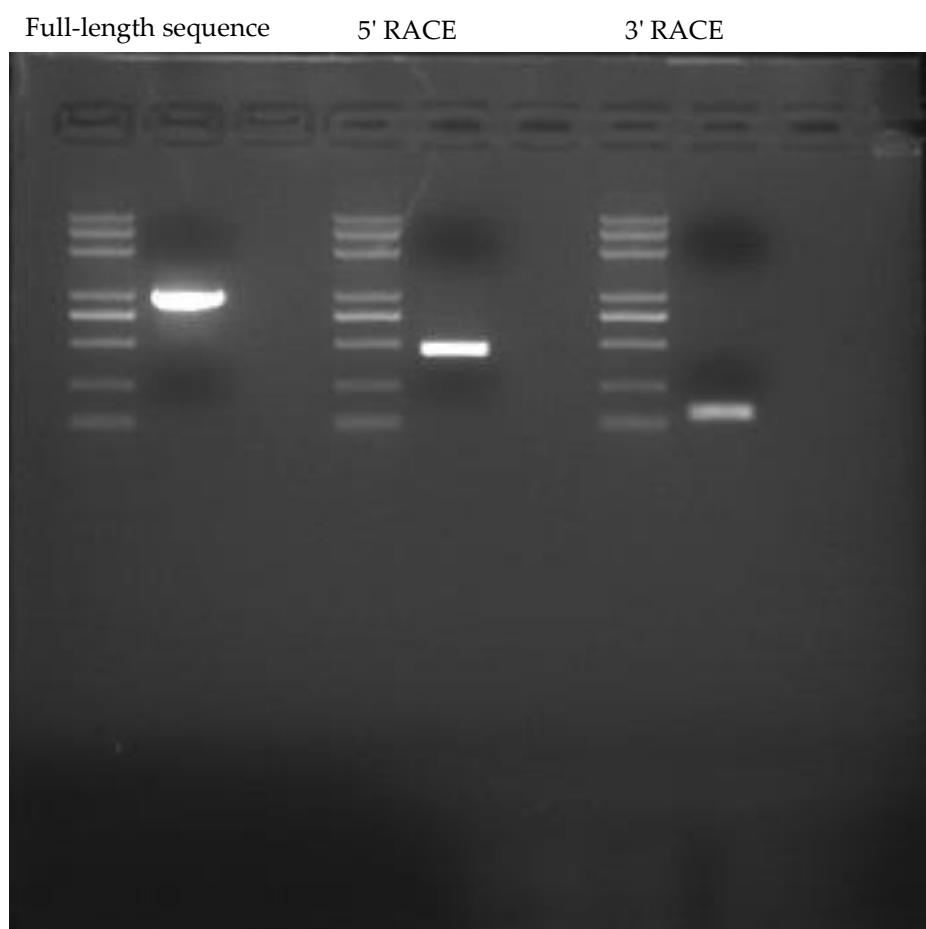

Supplement: Supplementary file 1 [file genes-14-01047-s001.zip › Original image of the gel.pdf]
